# Penile Reconstructive Surgery

**DOI:** 10.1155/2008/564032

**Published:** 2008-12-03

**Authors:** Miroslav L. Djordjevic

**Affiliations:** Pediatric Urology Department, University Children's Hospital, Tirsova 10, 11000 Belgrade, Serbia

Penile reconstructive surgery has always been a notable challenge in the field of genitourinary reconstruction because of the fact that it attaches huge importance to the functional and aesthetic aspects of this organ and the genital region as a whole. During the last decades, many operative procedures and their modifications for the treatment of both congenital and acquired anomalies are published. Based on the established principles of reconstruction, as we strive forward in this difficult art, we also feel the need to adapt to growing demands of rewriting the stiff challenges of techniques, their modifications, the long-term results, and futuristic issues which encompass this subspeciality.

One of the most common congenital anomalies is *hypospadias*. Repair of this anomaly has become easier with Snodgrass principle for distal and buccal mucosa graft combined with genital flaps for proximal forms. However, complication rate is still not minimized to be satisfied. Heidelberg group in the article entitled “Hypospadias” with their vast experience had discussed all aspects of hypospadias in details as it stands today commencing from its understanding as a developmental anomaly to its presentation, preparation, operative techniques as they stand today based on well defined historic and current concepts. Their overview emphasizes the fate of urethral plate in various distal and proximal surgical methods algorithmically to bring about satisfactory planning and execution of each treatment arm. The authors have in great details described their preferred approach of longitudinal preputial or penile flap in both distal and proximal hypospadias and presented its visual and schematic understanding.

Reinberg et al. in “Tunneled tunica vaginalis flap for recurrent urethrocutaneous fistulae” in a retrospective clinical analysis present their 5-year data on a set of 12 boys who underwent tunneled tunical vaginalis flap repair for recurrent fistulae in all locations. Another group from Detroit have presented a unique group of patients in their 5-year study where they discuss a subset of “iatrogenic strictures” presenting in adulthood resulting from childhood hypospadias surgeries. The complexity of these cases is increased manifold due to reasons of delayed presentation of many decades, varying lengths, as well as poor healing capacity due to scarred territory from previous surgical attempts and associated complicating factors of renal failure and fistulae.

The current management of patients with *intersex*, now named as disorders of sex development, presents results of updated etiological and outcome data as well as refined surgical procedures, as discussed in “Adult urethral stricture disease after childhood hypospadias repair.” Ambiguous genitalia play a role in gender differentiation, and surgical treatment should give answers for psychosexual questionnaire of this population. Gupta et al. in “Male genitoplasty for intersex disorders” have come up with a large series review to discuss dilemmas in gender reassignment in intersex disorders in the childhood age group. In their review which includes majority of cases of proximal hypospadias, authors advocate early and complete chordee correction in childhoood for satisfactory phallic growth. The issue of gender reassignment is a delicate one in paediatric age group and needs to take into consideration the community, parental wishes, the state of child, and its phenotypic gender of being reared, as well as the age of presentation. In masculanising genitoplasty satisfactory hormonal treatment, removal or preservation of mullerain structures and parental counselling are key features. It involves a dedicated teamwork and close yet longterm followup for corrections with changing age and hormonal management.

The degree of curvature, the type of deformity, erectile dysfunction, and penile length are all characteristics that are assessed in choosing the best surgical intervention in *Peyronie's disease*. The majority of procedures are usually followed with some loss of length. Using radical geometrical principles in creating and fashioning the graft with appropriate size leads to precise correction with penile lengthening. The first review from Aboseif et al. “Review of the surgical approaches for Peyronie's disease: corporeal plication and plaque incision with grafting” judges our understanding of surgical approaches to date in managing Peyronie's plaque—from plication to grafting. Their review article focuses on surgical evolution and compares the two methods from all recent publications thus judging the advantages of both techniques. A rationale approach today would be proper counseling of each well-informed patient and a surgeon capable of the armamentarium of techniques to choose from as required in an individual case with intraoperative decisions which hold the key. More complex and multiple deformities would benefit with a combination of the techniques described to ensure complete correction.

In another paper, “Peyronie's reconstruction for maximum length and girth gain—geometrical principles,” Egydio et al. redefine their concept of straightening by geometrical principles in yet another landmark study of 521 treated patients over 8 years who achieved satisfactory sexual health with the performed straightening and enlargement technique divided into two arms of use of bovine pericardial grafts and the corresponding arm with additional penile prosthesis. A small subgroup of patients with grafting alone who developed deterioration of erectile function in postoperative followup were treated with penile prosthesis to satisfactory outcomes. The penile disassembly described earlier is a huge assistance in exposing even the most distal deformities for complete correction. As we strive toward the search of an ideal graft for penile reconstructive surgeries, the use of bovine pericardial grafts with its advantages as demonstrated in such large series safely gives confidence of yet another biocompatible graft with long-term results. Prospective comparative studies with other contemporary surgical techniques would go a long way in furthering our knowledge toward ultimate patient satisfaction in Peyronie's disease.

In contrast, Piacentini et al. in “Preservation of cavernosal erectile function after soft penile prosthesis implant in Peyronie's disease: long term follow up” present an excellent retrospective review of intermediate results of 6 years in 12 patients who have been treated with semirigid prosthesis alone for Peyronie's disease without any plaque treatment. Their study is based on the hypothesis which heavily relies on the residual cavernosal tissue that becomes a peripheral component to the prosthesis and plays a healing role in further straightening as well as being available for future pharmacologic therapy.

A wide variety of medications, devices, and surgical interventions are available for patients with *erectile dysfunction.* Technological improvement of penile prosthesis lead to their rising popularity, but strict indications and rigorous surgical principles for implantation should always be respected (followed) in order to avoid unnecessary surgery and possible complications with severe psychological consequences. The next exciting section of this issue is devoted to the difficult art and contemporary world of penile prosthesis. Aboseif et al. in “A preliminary report on combined penoscrotal and perineal approach for placement of penile prosthesis with corporal fibrosis,” in a comprehensive retrospective study of 15 patients, review their state-of-the-art techniques for penile reconstruction in difficult penile prosthesis placement. Their group is divided into two treatment arms of severe Peyronie's tunical fibrosis and the other of severe tunical fibrosis of previous prosthesis-related complications. The data are very interesting and larger prospective studies for longer duration could throw more light to our continued search for ideal techniques. In another review, “Penile corporeal reconstruction during difficult placement of a penile prosthesis,” they discuss their preliminary results in combined penoscrotal and perineal approach for complex corporal fibrosis spanning 3 patients. Finally, Bettocchi et al. in “Penile prosthesis: what should we do about complications?” made a review of penile prosthesis implantations, indications, and, especially, a discussion about possibilities in the treatment of complications.

Last but not least, *penile carcinoma* is an aggressive disease with significant treatment-associated psychosexual morbidity. Despite high control rates with radical surgical approaches, organ-sparing surgery should be considered to achieve better psychosexual life. Lisbon group in “Organ preserving surgery for penile carcinoma” brought forth the idea of balance between the functional and anatomical aspects of this organ weighing against any compromise with local oncolgical radicality in this malignancy. They explore all possibilities of penile preserving approaches in penile cancers by various operative and nonoperative interventions in properly selected patient with a word of caution that the approach to organ preservation can be applied to low-grade and low-stage tumours until more evidence emerges from prospective studies. To further attempts at proper organ preservation techniques, the recent description of penile disassembly and excision—reconstruction would go a long way in achieving satisfactory results.

Finally, Hoebeke et al. in “Reconstructive surgery for severe penile inadequacy: phalloplasty with a free radial forearm flap or a pedicled anterolateral thigh flap?” have summarized their experience on the devastating condition of severe penile inadequacy in young males. Phalloplasty remains a challenge in reconstruction and is the hallmark achievement in transsexual surgery and severe penile deficiency that results from trauma, micropenis, and penile amputations. This series describes the procedure and consequences of 11 young males who underwent radial forearm flap or anterolateral thigh flap neophallus reconstruction.

*Miroslav L. Djordjevic*

